# A qualitative assessment of health extension workers’ relationships with the community and health sector in Ethiopia: opportunities for enhancing maternal health performance

**DOI:** 10.1186/s12960-015-0077-4

**Published:** 2015-09-30

**Authors:** Maryse C. Kok, Aschenaki Z. Kea, Daniel G. Datiko, Jacqueline E.W. Broerse, Marjolein Dieleman, Miriam Taegtmeyer, Olivia Tulloch

**Affiliations:** Royal Tropical Institute, P.O. Box 95001, 1090 HA Amsterdam, The Netherlands; VU University Amsterdam, Athena Institute for Research on Innovation and Communication in Health and Life Sciences, De Boelelaan 1081, 1081 HV, Amsterdam, The Netherlands; REACH Ethiopia, P.O. Box 303, Hawassa, Ethiopia; Department of International Public Health, Liverpool School of Tropical Medicine, Pembroke Place, Liverpool, L3 5QA, UK

**Keywords:** Health extension workers, Community health workers, Maternal health, Ethiopia, Performance, Community involvement, Trust

## Abstract

**Background:**

Health extension workers (HEWs) in Ethiopia have a unique position, connecting communities to the health sector. This intermediary position requires strong interpersonal relationships with actors in both the community and health sector, in order to enhance HEW performance. This study aimed to understand how relationships between HEWs, the community and health sector were shaped, in order to inform policy on optimizing HEW performance in providing maternal health services.

**Methods:**

We conducted a qualitative study in six districts in the Sidama zone, which included focus group discussions (FGDs) with HEWs, women and men from the community and semi-structured interviews with HEWs; key informants working in programme management, health service delivery and supervision of HEWs; mothers; and traditional birth attendants. Respondents were asked about facilitators and barriers regarding HEWs’ relationships with the community and health sector. Interviews and FGDs were recorded, transcribed, translated, coded and thematically analysed.

**Results:**

HEWs were selected by their communities, which enhanced trust and engagement between them. Relationships were facilitated by programme design elements related to support, referral, supervision, training, monitoring and accountability. Trust, communication and dialogue and expectations influenced the strength of relationships. From the community side, the health development army supported HEWs in liaising with community members. From the health sector side, top-down supervision and inadequate training possibilities hampered relationships and demotivated HEWs. Health professionals, administrators, HEWs and communities occasionally met to monitor HEW and programme performance. Expectations from the community and health sector regarding HEWs’ tasks sometimes differed, negatively affecting motivation and satisfaction of HEWs.

**Conclusion:**

HEWs’ relationships with the community and health sector can be constrained as a result of inadequate support systems, lack of trust, communication and dialogue and differing expectations. Clearly defined roles at all levels and standardized support, monitoring and accountability, referral, supervision and training, which are executed regularly with clear communication lines, could improve dialogue and trust between HEWs and actors from the community and health sector. This is important to increase HEW performance and maximize the value of HEWs’ unique position.

## Background

In 2004, the Government of Ethiopia introduced the Health Extension Programme (HEP), a free primary health care package with four components: disease prevention and control, family health, hygiene and environmental sanitation, and health education and communication. A female cadre of salaried community health workers (CHWs) called health extension workers (HEWs) was introduced nationally. HEWs are secondary school graduates and receive a 1-year training in basic health service delivery^1^. They are selected from the communities that they serve and are supposed to work at the health post level for 25% of their time and in the community for the remaining 75% [1–4]. Over 38 000 HEWs are employed in Ethiopia, contributing to a significant increase in health service coverage in recent years [2].

A clearly defined hierarchy links the health sector and HEWs to the community. The *woreda* (district) health office has general oversight of the health system. One health centre is, on average, linked to five health posts and together they form the primary health care unit. A health post serves a population of about 5000 and is staffed by two HEWs who are technically and administratively accountable to health centres [2]. Health professionals from the health centres supervise HEWs, and the HEWs refer clients in need of higher level health care to health centres or hospitals [5]. HEWs are accountable to the administration of the *kebele* (lowest administrative unit), who in turn are responsible for giving support to the HEWs [5].

**Figure 1:**
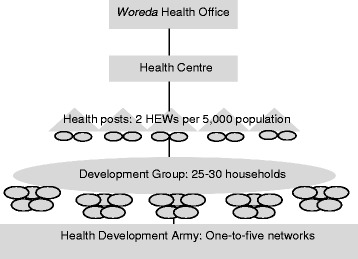
**Overview of HEWs’ intermediary position between the community and health sector.**

HEWs are linked to the community through a network of community volunteers, who are members of the health development army (HDA). The HDA was introduced in 2012, officially replacing other community-based workers such as health promoters and traditional birth attendants (TBAs). It is based on gradual training of model families by HEWs. Model families become leaders of a group of five families known as the “one-to-five network”, who in turn form a “development group” of 25 to 30 households within a village. “Graduation” to a model family occurs after training in all components of the HEP and proven implementation at the household level. All members of the HDA are supposed to support HEWs in the implementation of the HEP [2,5]. An overview of HEWs’ intermediary position between the community and health sector is presented in Figure 1.

A number of the HEWs’ tasks are related to maternal health. These include provision of antenatal care, clean and safe deliveries^2^, postnatal care, family planning, immunization and nutritional advice. HEWs have contributed substantially to the improvement in women’s utilization of family planning, antenatal care and HIV testing [3]. However, their contribution to advocating for skilled delivery and conducting postnatal check-up seems much lower [3], and their knowledge and performance in maternal health-related tasks is poor [6]. Ethiopia’s maternal mortality ratio remains high: 676 deaths per 100 000 live births and only 10% of women deliver with a skilled birth attendant [7]. HEWs only conduct 1.6% of all assisted deliveries in the country [8]. There have been calls for improved performance of HEWs on maternal health-related tasks [2–4,6].

Evidence from CHW programmes worldwide has identified several factors, related to programme design, that can influence CHW performance. These include CHW task definition, human resource management (including training, supervision and incentives for CHWs), quality assurance processes, resources and logistics and CHWs’ links with the community and health sector [9,10]. Health systems are social institutions in which different actors are linked with each other in chains of relationships [11]. Strong interpersonal relationships between CHWs and clients (henceforth referred to as the community) on one side and CHWs and health professionals and supervisors (henceforth referred to as the health sector) on the other side are needed to ensure good CHW performance [12–16]^3^. It has been found that CHWs’ relationships with the community are strong when CHWs have been selected from and by their community and CHWs’ relationships with the health sector are strong when there is respect for the roles of CHWs from health professionals [9,10]. The importance of CHWs’ relationships for performance is accentuated by the nature of their work (CHWs as facilitators of community agency) and their intermediary position between the community and the rest of the health system. However, in-depth evidence is lacking on which factors hinder or facilitate relationships. We conducted qualitative research in southern Ethiopia to identify facilitators of and barriers to interpersonal relationships between HEWs and actors in the community and health sector and, where possible, their impact on HEW performance in maternal health.

## Methods

A qualitative study, using 14 focus group discussions (FGDs) and 44 semi-structured interviews, was conducted in 2013 in the Sidama zone of the South Nation Nationalities and Peoples Region of Ethiopia. We used qualitative methods in order to obtain in-depth insight into how relationships between HEWs and the community and health sector were shaped and, where possible, what made them facilitate or hinder HEW performance. The participants included HEWs, TBAs, health professionals and community members (Table 1). Participants were drawn from six *woredas* selected for a larger study, on the basis of diversity in maternal health performance and distance from the zonal capital. Study respondents were purposefully sampled to represent different ages and job experience and were identified with help of health centre and *woreda* health office staff.

**Table 1:** **Overview of focus group discussions and interviews**

**Method**	**Participants**	**No. per district**	**No. of districts**	**Total no. of respondents (total number of FGDs)**
Focus group discussions (FGDs)	HEWs	1	6	57 (6)
	Women in community	1	6	55 (6)
	Men in community	0 or 1	2	19 (2)
*Total*				*131 (14)*
Semi-structured interviews	HEWs	2	6	12
	Mothers	2	6	12
	Traditional birth attendants (TBAs)	1	6	6
	*Kebele* administrators	0 or 1	3	3
	Health centre heads or delivery case team leaders	1	6	6
	HEP coordinators	0 or 1	3	3
	Regional HEP coordinator	NA	NA	1
	Zonal HEP coordinator	NA	NA	1
*Total*				*44*

Data were collected by four local health systems researchers, who received a 1-week training in qualitative data collection for the purpose of the study. Semi-structured topic guides were developed in English, translated into Amharic and Sidamigna language and back-translated for consistency. The topic guides were piloted in an area that was not included in the study, and adaptations to questions were made. The FGDs and interviews included questions on demographic information, expected and performed tasks, career, experiences relating to maternal health, training, supervision, monitoring and evaluation and referral. The questions focused on barriers and facilitators with regard to these issues, including effects on HEW performance. Regarding relationships with the community and health sector, respondents were asked about all different actors with whom they interacted and whether relationships were strong or weak and why and how they facilitated or hindered their work. I nformation about performance of HEWs was self-reported and defined at two levels: the HEW level (this included self-esteem, motivation, attitudes, competencies, guideline adherence, job satisfaction and capacity to facilitate community agency as characteristics of performance) and end-user level (this included utilization of services, health-seeking behaviour, adoption of practices promoting health and community empowerment as characteristics of performance) [9]. Study participants gave informed oral or written consent. Daily debriefing sessions with all data collectors were held to discuss key findings, identify saturation of themes and refine lines of inquiry. All interviews and FGDs were digitally recorded, transcribed and translated into English. A sample of transcripts was randomly checked against the recordings by one researcher (AK).

The transcripts were independently read in pairs by four researchers to identify key themes and develop a coding framework. This process used open coding with regard to factors influencing relationships [17], combined with a pre-defined framework of factors that could influence performance [9]. Transcripts were coded using NVivo (v.10) software, emerging themes were discussed and the coding refined. The coded transcripts were further analysed and summarized in narratives for each theme and sub-theme. Study findings were presented, discussed and validated with the regional and *woreda* health offices in a stakeholder meeting.

The study was approved by the Royal Tropical Institute Ethical Review Committee in Amsterdam and the South Nation Nationalities and Peoples Region Health Bureau Research and Technology Transfer Core Process of South Ethiopia.

## Results

Interpersonal relationships between HEWs and actors in the community and health sector were influenced by several factors. First, programme design elements influencing HEWs’ relationships with the community are presented, followed by those influencing HEWs’ relationships with the health sector. Cross-cutting factors categorized as trust, communication and dialogue, and expectations (as summarized in Table 2), are presented throughout. These cross-cutting factors emerged as important influencers of relationships, within all identified programme design elements. Quotations are used to illustrate main themes.

**Table 2:** **Programme design and cross**-**cutting factors influencing HEWs**’ **relationships with the community and health sector**

**Programme design elements facilitating relationships**	**Cross**-**cutting factors influencing relationships**		
	**Trust**	**Communication and dialogue**	**Expectations**
HEWs’ relationships with the community			
Nature of HEWs’ position and role	HEWs being selected from the community that they will serve generally enhanced community trust in HEWs, partly facilitated by good attitudes and high self-esteem of HEWs as a result of serving their own community	HEWs residing in their community of service facilitated ongoing communication and dialogue with community members	
	If HEWs served a community which they were not originating from, community trust in them could be hampered		
Support for HEW activities from the community	Some HEWs were supported by TBAs, as both community and HEWs trusted the competencies of TBAs in child birth and related tasks above those of HEWs	Support from *kebele* administration, religious leaders and HDA leaders facilitated communication and dialogue between HEWs and community members, assisting HEWs in community mobilization, health education, identification of clients and referral; if this support was not present, communication and dialogue with community was hampered	Community expectations regarding TBA involvement were not always in line with the policy, and this created dilemmas, which could hamper HEWs’ relationships with community and TBAs
		In some areas, support from TBAs to HEWs was ceased, because of lack of communication between HEWs and TBAs if TBAs were still conducting deliveries, which is not allowed by government	
Community monitoring and accountability structures		Quarterly facility or public forums, political gatherings, *kebele* cabinets, pregnant women’s forums and community-based review meetings were structures that facilitated communication and dialogue between HEWs and the community, including feedback on performance	
HEWs’ relationships with the health sector			
Referral	Improper handling of referral cases hampered trust from HEWs and community in the health sector	Lack of referral forms and feedback after referral hindered communication between HEWs and the health sector	Community expectations with regard to payment of transport and higher level care did sometimes not match with the reality, hampering trusting relationships between HEWs (who made the referral) and community
Supervision		Supervision with a fault-finding approach and without feedback, partly as a result of lack of resources and training of supervisors, hindered communication between HEWs and supervisors/management	
Training			HEWs’ expectations regarding trainings and career advancement were not met, hampering relationships between HEWs and health sector
Monitoring and accountability structures		Irregular held monitoring and evaluation meetings hampered communication between HEWs and the health sector	
Support from other health professionals		Regular support from health professionals at health centre level enhanced HEWs’ competencies and made them feel part of a team	Sometimes, expectations from the management level about tasks of HEWs interfered with HEWs’ work

### HEWs’ relationships with the community

HEWs’ relationships with the community were facilitated by the following: the nature of HEWs’ position and role in the community, support from the community (including support regarding referral) and community-driven monitoring and accountability mechanisms.

#### The nature of HEWs’ position and role

Many respondents reported that the attributes shared by HEWs in the community assured a “natural link” between them and the community. Good relationships were reported to result from HEWs being selected from the community they are supposed to serve and continuing to reside in that community. Community members reported that HEWs being female was important to them, as they prefer to discuss maternal health issues amongst women. HEWs’ position as community members themselves appeared to safeguard trust in and respect for the HEW from the community side and a good attitude towards the community and enhanced self-esteem from the HEW side.

“First I trust God and then the HEW. She calls an ambulance when she finds a problem. We tell the HEW our problems. They are always with us.” (Mother, interview)

“…they [the clients] are our mothers as well, and we are serving our own community. Their children are our children, and the community is my community.” (HEW, interview)

In one *woreda*, a male respondent reported that when the selection system is not followed and HEWs do not come from the *kebele* they need to serve, their relationships with the community are constrained as a result of lack of trust from the community side, leading to poor performance.

#### Support for HEW activities from the community

##### Support from *kebele* and other leaders

Support from the community was demonstrated in various ways. Some HEWs reported that *kebele* administrators supported them in conducting home visits and maternal health education sessions. *Kebeles* are expected to facilitate pregnant women’s forums during which HEWs talk with all pregnant women in the *kebele*. HEWs and HEP administrators reported that these group discussions facilitated women supporting each other and assisted HEWs in conveying their antenatal health messages.

“We have the pregnant women’s forum with tea and coffee to discuss maternal health with them. This is not considered by other health offices, but we have taken the time in the forums to increase their participation and to discuss maternal health so that we help them and support them financially…” (HEW, FGD)

Other HEWs reported a lack of support from the *kebele* level, partly as a result of lack of *per diems* for activities related to health as compared to agriculture or education. This lack of support resulted in constrained communication and dialogue between HEWs and the community and lower motivation and job satisfaction, because of lower community attendance to health activities and meetings.

“…the kebele administration helped us after much negotiation and begging. Otherwise they wouldn’t support us on their own initiative…” (HEW, interview)

Some HEWs reported that besides *kebele* administrators, they involved religious leaders and elders to support their work regarding maternal health advocacy and communication with the community.

##### Support from the health development army

It was widely recognized that HDA leaders had been supporting HEWs in identification and referral of pregnant women, conducting postnatal care follow-up, mobilization of communities for immunization campaigns and health education in the community.

“We teach the women in our community. We, the leaders of the one-to-five network, give our advice to convince pregnant mothers. When their labour starts we call to the HEW to inform and conduct the delivery.” (Woman, FGD)

Most HEWs were positive about the role and functionality of the HDA, as it helped them with referral and advocacy tasks or had an impact on the community’s understanding of maternal health. Despite positive contributions by the HDA, the structure was inactive in some areas and some respondents reported that the voluntary nature of HDA work could constrain their potential.

##### Support from traditional birth attendants

The HEP currently promotes skilled delivery in health facilities, but HEWs are nevertheless supposed to be trained in conducting “safe and clean” deliveries in health posts. However, most health posts were found not to have provided delivery services in recent years, because of lack of skills, experience or confidence of HEWs, lack of materials and equipment, the traditional habit of home delivery with a TBA or bypassing of the health post by seeking delivery services at the higher level right away. One HEW raised the issue of expectations of community members exceeding her capability, which led to demotivation.

“They [the community] would like to give birth at the health post, but we tell them that training is done turn by turn and it will take some time to start the service at the health post … We are not giving the services which we are supposed to give.” (HEW, interview)

There is a national policy prohibiting TBAs to assist delivery; rather, they should focus on referral of women for skilled delivery. TBAs were, however, still found to conduct deliveries. The community, and sometimes also the HEW, trusted and preferred the TBAs conducting deliveries. This was related to good communication and dialogue (teamwork) between HEWs and TBAs but also lack of self-confidence, skills and competencies of HEWs in conducting deliveries, which until recently they were expected to perform as part of the HEP.

“…we call the TBAs to assist labour due to the skill gap and [low] confidence we have. …TBAs have stopped attending deliveries now, but because of a lack of skills we attend the deliveries with their help. We fear attending deliveries. …We call them and they help us.” (HEW, interview)

“People say ‘the known devil is better than the unknown God’, and the people believe in them [TBAs]. We also communicate with the TBA, because the TBA is more popular than me in the kebeles, so I use her to contact women.” (HEW, FGD)

Some TBAs reported difficult relationships with the HEWs as a result of TBAs conducting deliveries against the policy. They were excluded from activities managed by HEWs and were not invited for meetings. In other communities, the TBAs’ role was indeed restricted to referral. The tension between what communities and HEWs often preferred (TBA involvement) and what policy directed (TBAs restricted to referral role) created dilemmas for HEWs and weakened their potential as intermediaries between communities and the rest of the health system.

#### Community-driven monitoring and accountability mechanisms

The study identified several structures facilitating community monitoring and accountability. The performance of health centres was evaluated by the community during facility or public forums, held on a quarterly basis. At the health post level, HEWs were monitored by the *kebele* administration and sometimes by the leaders of the HDA. Many HEWs mentioned that they collected reports from the leaders of the HDA and incorporated them as part of their activities. Most HEWs stated that they had been conducting regular meetings with the HDA leaders, exchanging feedback on their work and receiving reports of activities performed by the HDA. This assisted HEWs to adjust maternal health education to the needs of the community.

“We meet every month with the leaders of the one-to-five network. We discuss our work, what is going on in the community; they also bring their report and discuss it.” (HEW, interview)

Other meetings used for discussing performance were s*hengo* (political gatherings), the pregnant women’s forums and *kebele* cabinets. Some HEWs reported that they evaluate the quality of the service they provide in the community during joint meetings with the *woreda* health office, *kebele* administration and community representatives. In this way, the monitoring and accountability structure is both related to the community and the health sector and there is enhanced communication and dialogue between all levels.

“Sometimes the community with the kebele administration gather and evaluate our performance… The kebele officials and the community give a witness about their satisfaction.” (HEW, interview)

### HEWs’ relationships with the health sector

HEWs’ relationships with the health sector were influenced by the following: referral, supervision, training, monitoring and accountability systems and support from other health professionals.

#### Referral

There is an established referral system between HEWs, health centres and hospitals, and all HEWs reported that they refer maternal cases when the situation is beyond their capacity. Referral was constrained by miscommunication between the health sector, HEWs and communities. Some HEWs used referral forms; however, most reported absence of referral forms at their health post. As a result, a HEP coordinator at the zonal level indicated that the referral record-keeping system was poor. Feedback from the referred facility to the HEW was variable. Some HEWs reported improper handling of referral cases in the health centre.

“The basic thing we have to consider is a woman should not die giving birth. Sometimes even death can happen in a health centre. I knew a woman died …, because the health centre didn’t refer her to the hospital as early as possible.” (HEW, FGD)

Lack of transport or requests for payments regarding transport for clients, requests for payments at the health centre level (which should be free) and fees for clients at the hospital level were reported. These constraints in the referral system further hindered HEWs’ relationships with the health sector and, because of this, their trusting relationship with the community. In some cases, HEWs got the blame of constraints that community members faced at the health centre or hospital level, as they were the ones referring the clients there.

#### Supervision

Supervision from the side of the health sector was mostly reported to be in place, although not always regularly implemented, sometimes caused by transportation problems. Some HEWs were satisfied; however, many complained about a fault-finding attitude of supervisors, an overemphasis on checking of records and registers and a lack of supportive and problem-solving approaches. Quotes from HEWs clearly show that adequate supportive supervision could increase their motivation and credibility.

“If the woreda supervisors come and see our work, we will be happy. We need encouragement from the woreda officials. We will be encouraged by the appreciation for our good work, but our morale will be affected if our good work is ignored.” (HEW, interview)

“What makes us not work hard is, when the woreda health office comes for supervision, they leave our strong parts and take very minor things and discourage us due to those things.” (HEW, FGD)

The responsibility for the direct supervision of HEWs recently changed from the *woreda* health office to the health centre, with a group of health professionals, each assigned to supervise and support one of the five health posts in the catchment area, forming the command post. They are expected to provide feedback to the *woreda* level. The majority of HEWs who participated in the interviews and FGDs stated that this recently introduced system was not yet functioning well. A lack of communication skills and knowledge related to the HEP among the HEWs’ supervisors was one of the weak points mentioned by some participants in the study that would limit the scope of supervisors to build the capacity of HEWs.

“Health professionals know the science very well but are not familiar with the health extension packages… The nurses who are more clinically competent are expected to give support to HEWs who know the packages very well: this is not logical.” (*Woreda* HEP coordinator, interview)

It was also mentioned that the health professionals who supported the HEWs were sometimes disrespectful or unfriendly to HEWs, leading to constrained communication, mistrust and demotivation. The majority of the HEWs interviewed mentioned that they did not receive written feedback after the supervision, which was confirmed by a respondent from one of the health centres. Few HEWs reported to receive feedback based on command post evaluation formats.

#### Training

Many HEWs reported disappointment with the limited possibilities for trainings. Refresher trainings on maternal health were reported to lack practical elements on delivery. Official trainings for upgrading were also a source of disappointment for many HEWs. The selection process was not clear, the entrance exams considered too difficult and promotion after attending the training was not guaranteed.

“Even if we get education opportunity and make improvements in our level, there is no difference to me. Because the HEW who upgrades her status will again be assigned in the [same] kebele, no transfer is given to her, just as if she had not joined the school.” (HEW, FGD)

On-the-job training from the health centre was supposed to take place once a week, but often, HEWs reported that this was not happening.

#### Monitoring and accountability towards the health sector

The coordinator of the HEP at the regional level reported that the performance of HEWs was assessed during supervision and regular meetings.

We meet and contact HEWs directly when we provide supportive supervision at health post level, when we provide refresher trainings. During these times we conduct discussions on health work performance and build their capacity…” (Regional HEP coordinator, interview)

However, from the perspective of HEWs, these supervision meetings were not always held or conducted in a supportive way. HEWs’ work is based on monthly plans. Reports, containing information from the health management information system and data collection from various programmes, were supposed to be sent from the health post to health centre and from there towards the *woreda* health office. HEWs were supposed to have weekly meetings with the command post, monthly meetings with the health centre and quarterly meetings with the *woreda* health office to discuss these reports, but HEWs and health centre staff reported that meetings were irregular.

#### Support from other health professionals and managers

HEWs had regular contact with health professionals at the health centre level, which was important to enhance their competencies and made them feel part of a team. Many HEWs and health centre respondents reported good relationships between HEWs and health professionals, who supported HEWs on special occasions or regarding specific services.

“They help us very well during the vaccination mobilization period.” (HEW, FGD)

“To link health posts with health centres, starting from last year, all staff from health centres provide support to health posts once or twice per week, to identify gaps and to deliver services together with HEWs, especially during antenatal care services to run HIV tests since HEWs cannot do this…” (*Woreda* HEP coordinator, interview)

Some HEWs reported competing programmes and expectations from the upper level, intervening with their planned activities.

“We may plan to accomplish certain activities, but from the woreda health office we will be told to do other things… When we plan to teach mothers or want to have community conversations, the woreda health office may tell us to do other activities like vaccination campaigns.” (HEW, FGD)

Not only were HEWs expected to conduct health-related tasks beyond those scheduled according to their work plan, some respondents also reported HEWs’ involvement in other sectors and politics, requested by administrators. This disturbed their regular work, led to high workload and in certain cases to mistrust from the community towards the HEW.

“Sometimes we are involved in the activities coming from women affairs and the education sector. We are also involved in political matters. We are quarrelling many times with people about these things. If we are not involved in these activities, they cut our salary.” (HEW, interview)

## Discussion

**Figure 2:**
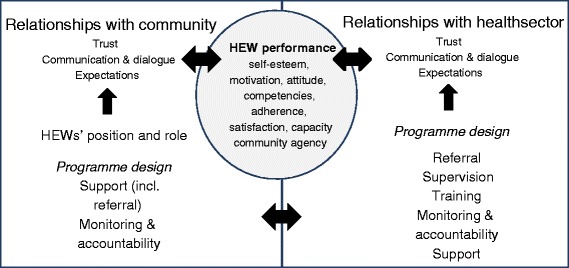
**Factors influencing relationships between HEWs and the community and health sector and the influence on HEW performance.**

HEWs have a unique intermediary position between the community and health sector, which gives them the ability to act as brokers and facilitators of dialogue and trust [18,19]. To be able to perform optimally, HEWs require strong interpersonal relationships with actors in the community and health sector. Programme design elements related to support and accountability either facilitated or hindered relationships between HEWs and the community or health sector. Trust, communication and dialogue and expectations (of actors in the community, health sector and HEWs themselves) were cross-cutting factors influencing relationships. The quality of relationships was, in some cases, reported to influence HEW performance at the individual level, in particular motivation. An overview of factors influencing relationships between HEWs and the community and health sector, and thus influencing HEW performance, is presented in Figure 2.

### Tensions of HEWs’ intermediary position

The natural position and role of HEWs in their *kebele* safeguards trust, credibility and respect towards the HEW and their engagement with that community, which can enhance HEW performance [13,14,20,21]. However, HEWs are selected by the health sector and must meet the standards of the sector. There are sometimes differing expectations from the community and health sector regarding roles and tasks of HEWs, for example, regarding HEWs’ role in childbirth or involvement in political matters, leading to tension (when HEWs feel they cannot meet expectations [22]), high workload and demotivation. Clearly demarcated roles and tasks of HEWs which are communicated with all levels using job descriptions, government directives and explanations in joint meetings where community and health sector representatives are present could prevent this. This should be taken into account in current debates, in which HEWs are increasingly expected to advocate referral to skilled deliveries in health centres or hospitals rather than assisting “clean and safe” deliveries (conducting deliveries themselves) in the community.

HEWs’ position as intermediaries enables support from both the community and health sector towards the HEW, which could enhance HEW performance [9]. Community support generally relied on voluntary systems, while the formal community structure (the *kebele* administration) seemed to lack leadership when it comes to supporting health services. Reinforcement of the support from the *kebele* administration towards the HEP is recommended to increase HEWs’ credibility, ability to initiate communication and dialogue with communities and motivation. Support from TBAs was reported as well, although this sometimes presented problems for HEWs, because relationships between HEWs and TBAs did not always correspond with a TBA’s new roles as directed by the government. Relationships between HEWs and TBAs were directed to be focused on referral, and the collaboration between HEWs and TBAs in conducting deliveries can thus be seen as an unintended effect of their relationship. Thus, HEWs were not always working in line with the health system’s standards as a result of tensions emerging from their intermediary position. HEWs sometimes felt they should support well-respected TBAs who were still conducting deliveries, thereby accommodating views of the community to maintain trust and keep good relationships with the community.

HEWs’ linkage with the health sector through referral was identified but was not always strong, because of communication problems and sometimes a lack of trust of communities and HEWs regarding the costs and quality of higher level services. Improvements regarding handling of referred cases, payments and feedback could improve performance. The HEW supervision system had recently changed which led to unclear roles and insufficiently trained supervisors. Supervision meetings with top-down communication or a fault-finding nature can hinder the trust of health workers in the health sector and hamper their performance [23,24] and their relationships with the community [25]. HEW supervisors are predominantly engaged in clinical activities, making training on supportive approaches to supervision, preventive and community health necessary to capacitate them in supervision of HEWs. Furthermore, supervision time should be officially allocated, as health centre staff can be overloaded with other work. Possibilities for peer-based approaches in addition to the current supervision system could be considered, as CHW peer support groups were found to improve performance in Rwanda [26]. Refresher training could establish relationships with other health workers or enhance trust from other health workers because of the upgraded knowledge of HEWs. However, HEWs were generally dissatisfied with their opportunities for receiving trainings. Clear selection processes for training attendees [24,27] and clear prospects of possibilities for upgrading after training are needed to keep HEWs motivated and prevent attrition [13,28]. Visible supervision and training of HEWs by the health sector is important to enhance credibility of and trust in HEWs, as found in other settings [29–32].

#### Relationships between HEWs, community and health sector enhancing performance

Community support to HEWs in Ethiopia has been demonstrated by voluntary CHWs^4^, churches, mosques and community associations [12,33]. The new HDA structure presents an opportunity for further strengthening HEWs’ relationships with the community side, in that it provides actual support towards HEWs’ tasks, monitoring of HEWs’ performance and accountability. Dysfunctional or inactive community structures have been reported to negatively influence CHWs’ embedment in and communication with the community and CHW performance in other settings [24]. Other studies have shown the effectiveness of community monitoring in promoting the performance of professional health workers [34] and CHWs [35,36]. However, more research is needed on the exact mechanisms of how this can improve the performance of CHWs [37]. HEWs felt that relationships with religious leaders and elders were important to enhance their performance. This facilitating relationship has also been observed in other studies [12,38,39]. HEWs and HDA leaders are all female; this was found to be positively valued by the community, because of the cultural suitability of handling reproductive health issues by women. This has been found in other settings as well [40–42]. However, more research would be needed to establish if the gender of HEWs could negatively influence their relationships with traditional leaders, husbands of pregnant women and other male community members and so hinder HEW performance in maternal health.

The command post as the monitoring and accountability system at the side of the health sector seemed to be functional in some areas and needs further scale-up. Evaluation meetings with community and health sector representatives were held in some areas. This could be a vehicle for improved communication and accountability towards both sides and needs further expansion and investigation.

#### Health systems as social institutions

Health systems are social institutions with chains of relationships between different actors. Optimal performance depends on the strength and nature of relationships between all actors [11]. We explored the relationships between health professionals and HEW supervisors, HEWs and their communities. The influence of relationships on HEW performance was reciprocal: HEW performance could also influence trust, communication and dialogue and expectations (Figure 2). For example, HEWs’ lack of competence in childbirth could negatively affect the trust of the community in the HEW and thereby hamper relationships between the community and HEWs. When we see health systems as social institutions, the ways of bringing about change in health systems go beyond altering written rules and distributing resources and extend to effectively managing relationships between different actors [43]. HEWs’ relationships with the health sector could be strengthened by human resource management practices and approaches that focus on building trust and improving dialogue within the workplace, such as problem-solving supervision and culturally appropriate communication [11,23,25]. Improved relationships between HEWs and the health sector could positively influence their relationships with the community through improved trust and motivation, which could further positively influence HEW performance. In addition, programme designs that facilitate community support and monitoring and accountability could further improve trust, communication and dialogue between HEWs and the community and manage expectations at all levels, which in turn could enhance HEW performance.

#### Study limitations

This study is limited by several factors. Firstly, the study was part of a broader research project that included all factors that could influence HEW performance. Issues related to HEWs’ relationships with the community and health sector were derived from this broader research, and thus, some in-depth questions probing on those relationships were not asked. However, we think that the data presented in this article are representative for the six districts included in the study, as interpersonal relationships emerged as one of the most important influencers of HEW performance in the data set. Secondly, as in any qualitative study, one must contend with social desirability bias. We tried to avoid this by in-depth probing and conducting the interviews and FGDs in neutral environments. Thirdly, the outcomes of this study cannot easily be generalized to other settings. However, by including respondents from different settings and by triangulation via different types of respondents and data collection processes, the findings do present useful insights for other settings. Lastly, the study focused on relationships between HEWs and the community and health sector. Relationships among HEWs and between HEWs and other community-based workers were not fully assessed, although they could influence HEW performance, as presented in other studies from Ethiopia [44–46]. Furthermore, relationships are also influenced by more personal characteristics of HEWs.

## Conclusion

This study provides in-depth information on which factors hinder or facilitate relationships between HEWs, the community and health sector, which can inform other CHW programmes aiming for enhanced CHW performance. We found several programme design elements that could facilitate interpersonal relationships of HEWs with actors from the community and health sector, especially related to support of and accountability to both sides. Within those programme design elements, trust, communication and dialogue and expectations were influencing the strength of relationships. Clearly defined roles and responsibilities at all levels and standardized support, monitoring and accountability, referral, supervision and training could improve communication, dialogue and trust between HEWs and actors from the community and health sector. This is important to maximize the value of HEWs’ unique intermediary position and ultimately improve HEW performance, not only in maternal health but regarding their roles and tasks in all components of the HEP.

### Endnotes

^1^Our study is based on the following definition of CHWs: “health workers performing functions related to health care delivery; who have received a limited training focused on activities they need to carry out in the context of the intervention(s) they implement; and have received no formal professional or paraprofessional certificate or tertiary education degree” [47]. Therefore, HEWs are seen as CHWs. However, compared to CHWs in other countries, HEWs may be seen as a semi-professional or auxiliary cadre.

^2^Clean and safe deliveries are conducted by the HEW at the health post level. They cannot be referred to as skilled deliveries, which are conducted by skilled attendants in health facilities (health centres and hospitals).

^3^The community, HEWs and health sector together form the health system.

^4^Volunteer CHWs were present in the past; officially they are replaced by the HDA.

## Abbreviations

CHW: Community health worker
FGD: Focus group discussion
HDA: Health development army
HEP: Health Extension Programme
HEW: Health extension worker
TBA: Traditional birth attendant
